# Medical Students Abroad

**Published:** 1858-05

**Authors:** 


Medical Students Abroad.—In London, during the past session, the number of students was 1050. In Paris, 1027 took out inscriptions for 1857–58. In Dublin, the whole number was 573; and we find in the January number of the *Glasgow Medical Journal* that upward of 400 students were attending the classes at the University of Glasgow and Anderson's University; thus showing an increase over their last session.

